# Quantifying responses to changes in the jurisdiction of a congestion charge: A study of the London western extension

**DOI:** 10.1371/journal.pone.0253881

**Published:** 2021-07-01

**Authors:** Laila Ait Bihi Ouali, Davis Musuuga, Daniel J. Graham

**Affiliations:** Dept. of Civil and Environmental Engineering, Imperial College London, London, United Kingdom; The University of Tokyo, JAPAN

## Abstract

This paper quantifies behavioural responses to changes in the jurisdiction of a congestion charge, with a successive focus on (i) an extension and (ii) a reduction in the size of the charging zone. We exploit the unanticipated nature of both the implementation and removal of London’s Western Expansion Zone (WEZ) as quasi-natural experiments to test whether individual responses to policies are asymmetric. We use the UK Department of Transport Annual Average Daily Flow (AADF) data, which records traffic flows for seven transport modes (including cars, buses, bicycles, heavy and light goods vehicles). Using a difference-in-differences approach, we find that the introduction of the WEZ led to a 4.9% decline in road traffic flows in the new congestion charge area. These results are robust to different model specifications. HGVs traffic did not significantly change post-WEZ, which indicates that their road demand is price inelastic. The removal of the WEZ led to no significant variations in traffic. This result indicates asymmetry in behaviour with persistent changes in post-intervention traffic demand levels.

## 1 Introduction

Congestion-related economic and welfare costs are substantial and have gone up in recent decades. In the US, they have doubled since 2000 to reach an estimated $166 billion in 2017 [1]. In addition to causing economic losses through lost times, congestion can damage the environment and human health via increased vehicle emissions [2, 3]. These vehicle emissions include Nitrogen oxides (*NO*_*x*_) and particulate matter, which have been linked to increased child mortality and premature births [4, 5]. Negative effects associated with congestion have prompted governments to intervene by either increasing capacity or implementing demand management tools such as taxes, driving restrictions and subsidies. However, these policies can result in increased traffic volumes through induced demand, which limits their efficiency [6, 7].

Since its first introduction in Singapore in 1975, congestion charging has been trialled in various cities including Stockholm, Gothenburg and London [8]. The underlying principle of congestion charging is to internalise the social costs of using the road in the price. Following that principle, each road user should be charged for the disbenefit caused by their contribution to congestion which results in considerable social welfare improvements [9, 10].

Previous congestion charge scheme studies mostly assess the responses to their introduction: most empirical analyses show that congestion charging is associated with a decline in both congestion, traffic volumes, accidents and air pollution [11–15]. However, congestion charge removals have received much less attention in the literature. Some studies find that congestion charge removals were followed by a “hysteresis effect”, *i.e.* individuals go back to the way they used to travel once the program ceases to exist. In the cases studied, *e.g.* the Stockholm 2006 trial [12, 16], this hysteresis effect is likely to have been observed because the congestion charge was quickly removed after its implementation. However, these local studies do not use causal inference methods, and mostly follow a general travel framework [17–19].

To our knowledge, there are currently no papers jointly studying the implementation and removal of a congestion charge yet. The present paper aims to bridge that gap and to contribute to the literature by providing a complete analysis of the different stages of a congestion charge scheme. This enables us to test whether the implementation and removal of a congestion charge have symmetric effects, which is crucial in informing policymakers on the consequences of alterations that can be made to these programs. In analysing responses to the implementation and removal of a congestion charge scheme, we also can understand both the immediate short-run effects of the congestion charge and the long-lasting effects of road pricing.

It is not possible to know *a priori* responses to the withdrawal of a congestion charge. Two opposite effects are at play: firstly, the removal of the congestion charge reduces the costs of travelling by car, which incentivises car use and can, in turn, increase traffic volumes. However, the presence of the congestion charge for a certain amount of time in London is likely to induce a salient decrease in driving habits as individuals shifted away from using cars to avoid additional costs since the scheme started. Therefore, in the long run, changes in habits could present themselves in the form of a decrease in car ownership. Previous studies have shown that the long-run effects of pricing tend to outweigh the short-run effects [20], which highlights that transport decisions triggered by financial disincentives can evolve over time. Therefore, it is crucial to test whether the reduction in the costs of travelling by car mentioned above are a substantial financial incentive to travel by car once habits have shifted after years of car ownership disincentives.

Another contribution of the present analysis is in the choice of the identification strategy. In this paper, we use causal inference methods which allow us to quantify behavioural responses to changes in the congestion charge scheme and enable us to pinpoint precisely individuals’ channel of response.

In this study, we analyse responses to the introduction and the removal of the Western Expansion Zone of the London congestion charge. The initial London congestion charge zone was expanded westwards in 2007 and then removed in 2010 following successive political shifts. The unanticipated nature of these events makes them suitable quasi-natural experiment candidates [21]. This enables us to quantify responses to road traffic management policies in terms of traffic flow. We perform separate analyses to uncover differentiated responses by transport modes. Our identification strategy uses a causal inference methodology (difference-in-differences estimation, DiD) which allows us to quantify behavioural responses cleared of time and location effects. We use the United Kingdom’s Department for Transport (DfT) Annual Average Traffic Flow (AADF) panel data for Greater London. AADF data record traffic flows for seven transport modes: cars and taxis, buses and coaches, heavy goods vehicles (HGVs), light goods vehicles (LGVs) and motorcycles. This allows us to explore the causal effects of the WEZ successively on personal, commercial and public transport. Additional regressions exploit manual traffic count data for Greater London produced by the Consumer Data Research Centre (CDRC).

Our results show that the introduction of the WEZ led to a 4.9% decline in road traffic flows for all motor vehicles in the new congestion charge area. However, the traffic volume of HGVs did not significantly change post-WEZ, which implies their road demand is price inelastic. In contrast, we record no significant changes in traffic after the WEZ is removed. Results are robust to different specifications, to placebo tests and buffer tests. Therefore, estimates suggest that responses to the successive implementation and the removal of a congestion charge are not symmetric. After the implementation of a congestion charge, individuals are disincentivised to use their car in the new WEZ area as the costs of travelling by car in this zone increase. The lack of significant change post-removal seems to indicate that road pricing triggers long-run changes in travel behaviour that persist beyond the policy period. However, the withdrawal of a congestion charge increases financial incentives to use one’s car, yet this incentive might be marginal and insufficient to trigger a significant behavioural change.

The paper is structured as follows. Sections 2 and 3 review respectively the institutional background and the existing empirical literature on the CC. Section 4 describes our difference-in-differences method. Section 5 presents the data, and Section 6 presents the results, their discussion and robustness checks. Section 7 concludes.

## 2 Institutional background

Following a program of extensive analysis and planning [22, 23], the London congestion charge (CC) was first introduced in 2003. Its main official objectives were to (i) reduce congestion, (ii) improve journey time reliability, (iii) increase the quality of the bus system and (iv) provide income for the maintenance and development of the underground [24].

The original London CC zone used the Inner Ring Road as its boundary. The charge levied in 2003 amounted to £5 per vehicle between 07:00 and 18:30 every weekday (with the exclusion of public holidays) payable on the day either online, by phone or text message. Despite the possibility of getting weekly, monthly or yearly passes, discounts were not available. Failing to comply to the CC scheme led to a £80 penalty [25]. Discounted fees existed (*e.g.*, a 90% reduction in the fee for residents). Vehicle types exempted from the charge are the following: (i) motorbikes and mopeds; (ii) buses and coaches licensed in the DVLA ‘bus class’, (iii) London taxis; (iv) emergency service vehicles (*e.g.*, police vehicles, ambulances). In addition, Blue Badge holders, NHS staff and patients and fire fighters are also exempted from the CC fee. In addition, alternative fuel vehicles (*e.g.*, hybrid vehicles, vehicles over 3.5 tonnes that meet at least Euro III emissions standards, light commercial vehicles under 3.5 tonnes meeting emissions standards 40% above Euro IV standards) and electric vehicles had to pay only an annual £10 charge.

**Fig 1:**
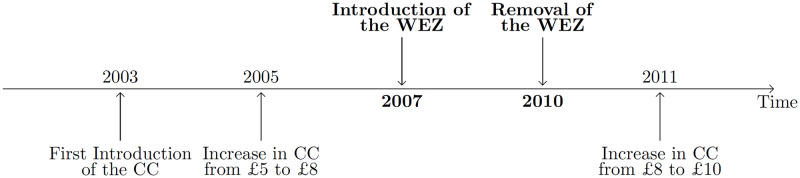
Timeline of the main geographical and pricing changes enforced to the London congestion charge scheme (source: [22]).

Since its first introduction, the London CC scheme has undergone several changes (see Fig 1 for a timeline), of which the most important is its westward extension. Plans to extend the central congestion zone westwards were first discussed in August 2004. The proposed extension into Kensington and Chelsea was deemed practical and workable as this area presents public transport alternatives for individuals and is also subject to continuous congestion [26]. In addition, charging hours were reduced by 30 minutes from 6:30pm to 6:00 pm. The WEZ covers an area of 17 km^2^, comparable in size to the original CC zone and includes most of the Westminster and Kensington and Chelsea boroughs. Note that detailed maps of the congestion charge area are available at:http://www3.westminster.gov.uk/docstores/publications_store/transportandstreets/western_extension_congestion_charging_leaflet.pdf. The WEZ operated as a single congestion zone with the rest of the initial London congestion charge zone, with a toll of £8 since 2005. London also had uncharged roads that provided an alternative for people who wished to avoid the tax and only wanted to cross central London rather than enter the zone.

The anticipated benefits of the extension were a 5 to 10% traffic volume reduction in charging hours alongside a 10–20% decrease in congestion [27]. Yet, this expansion was met with mixed reactions as some argued that the economic case for WEZ was weak [28]. The western expansion of the London CC zone became operational on February 19, 2007 and was removed on December 24th, 2010.

## 3 Literature review

### 3.1 Responses to the introduction of a CC scheme

A strand of literature studies the nature of responses to the implementation of a congestion charge [29]. To our knowledge, all of these studies find a significant decrease in traffic intensity in places where congestion charging is introduced. Traffic entering and leaving the London CC zone decreased by respectively 18% and 21% after its implementation [30] and that inbound trips to central London made by private cars reduced by 33% [24]. Other studies observing responses to the implementation of the WEZ find that the traffic reduced by 5.6% in roads within 5km of the WEZ boundary [31]. However, unlike our study, they do not control for time-varying borough characteristics which implies that responses might be subject to biases. In another WEZ study, the introduction led to a 14% reduction in four or more wheeled vehicles entering the zone (TfL, 2008) which is less than 30% attributed to the London CC scheme. Previous analyses suggest three reasons for this: (i) drivers who already pay the CC were not affected, (ii) the discount for residents increased the attractiveness to drive personal vehicles, and (iii) the WEZ has a greater proportion of residents who drive [32]. Responses are not only measured using traffic flows as outcomes and show that the London CC scheme led to a reduction in *NO*_*x*_ and other emissions in the CC zone and an increase in the surrounding areas [11, 13]. However, estimates still fail to fully capture the environmental effects of the CC as other factors (*e.g.*, advancements in engine technology) will also have affected pollution levels [33].

As mentioned previously, a significant decrease in traffic volume is unanimously observed after the implementation of a congestion charge. This implies that individuals respond in shifting their travel decisions towards other routes and towards other transport modes. After its initial implementation in London, the number of potentially chargeable vehicle kilometres reduced by 25% while the number of non-chargeable vehicle kilometres increased by 18%, which imply that individuals are incentivised to travel at earlier or later times in order to avoid paying the toll [34].

The implementation of a CC scheme also led to changes in the choice of transport mode, with studies showing that responses are of all the trips that were no longer made in central London, around half transferred to a form of public transport, a quarter avoided the CC zone, and 10% shifted to other forms of private transport [35]. In addition, the literature finds that combined effects on traffic volumes and public transport patronage enabled the CC to reduce congestion by around 30% during charging hours in the corresponding zone [35]. Other estimations indicate that the CC led to a 37% increase in bus passengers, half of whom were displaced from cars according to their estimations [30]. However, there has been a general increase in bus patronage across London [36], which might indicate that the aforementioned estimated effects might be overestimated. Other evidence nuances the aforementioned results by arguing that this response is likely to be a one off-shock [37]. Yet, all the aforementioned studies indicate that the WEZ led to substitution effects across transportation modes, with commuters shifting to other travel modes. Finally, the congestion charge implemented in Milan is found to lead to a major shift to other transport modes (such as motorbikes) which limited the positive impact of the policy [38].

This shift would ideally lead to a reduction in the volume of traffic and congestion. Descriptive statistics indicate that the WEZ and the area surrounding it experienced an increase in congestion; however, they could be attributed to road works which effectively reduced road capacity [39]. This result underlines the necessity to use causal inference tools to measure and precisely quantify responses to the implementation of a congestion charge in a new area.

### 3.3 Responses to the reduction and/or withdrawal of a CC

Most studies assess responses after a CC scheme is implemented. However, less attention has been paid to the reduction and/or the complete removal of a congestion charge. Overall, studies of responses to the removal of a CC indicate that the duration of the removal and whether it is permanent or temporary affect the nature of responses. Responses to removals appear to be milder than responses to the implementation of a CC.

Studies of responses to the successive implementation and withdrawal of the six-month CC trial carried out in Stockholm find that traffic reduced by about 22% during the trial and then increased almost completely back to its original pre-trial level after the removal and remained only slightly lower than the pre-policy stage [40]. They attribute this slight reduction in traffic post-trial to major roadworks on a central bridge, which leads them to the conclusion that introducing and removing a CC induces symmetrical responses. However, this trial only lasted for less than a year, and individuals also knew that this trial was temporary, implying that this policy did not last long enough to alter travel behaviour determinants (*e.g.*, car ownership), leading individuals to return to their previous travel habits once the trial ended.

A congestion charge was implemented since 2008 in Milan, but was then suspended for 50 days in 2012. This event is a good candidate for a quasi-experimental analysis as it was unanticipated and happened for political reasons. Moreover, the temporary suspension had no significant effect on the overall traffic flow [38]. Yet, they find a shift in travel modes as the flows of polluting cars increased whereas flows of hybrid and electric cars decreased. This indicates that a short-term removal of a congestion charge is not likely to lead to neither important responses nor symmetrical to the implementation of a congestion charge.

Similarly in Trondheim, the toll cordon was removed in 2005 after 15 years of operation, providing an opportunity to analyse responses to a permanent withdrawal of a congestion charge. This removal led to a 12% increase in traffic during charging hours as commuters moved their trip times back to the former charging hours [41]. This is evidenced by an additional 10% decrease in traffic over uncharged hours. These results imply that permanent CC removals lead to more substantial changes in behaviour than temporary ones.

## 4 Method

**Fig 2:**
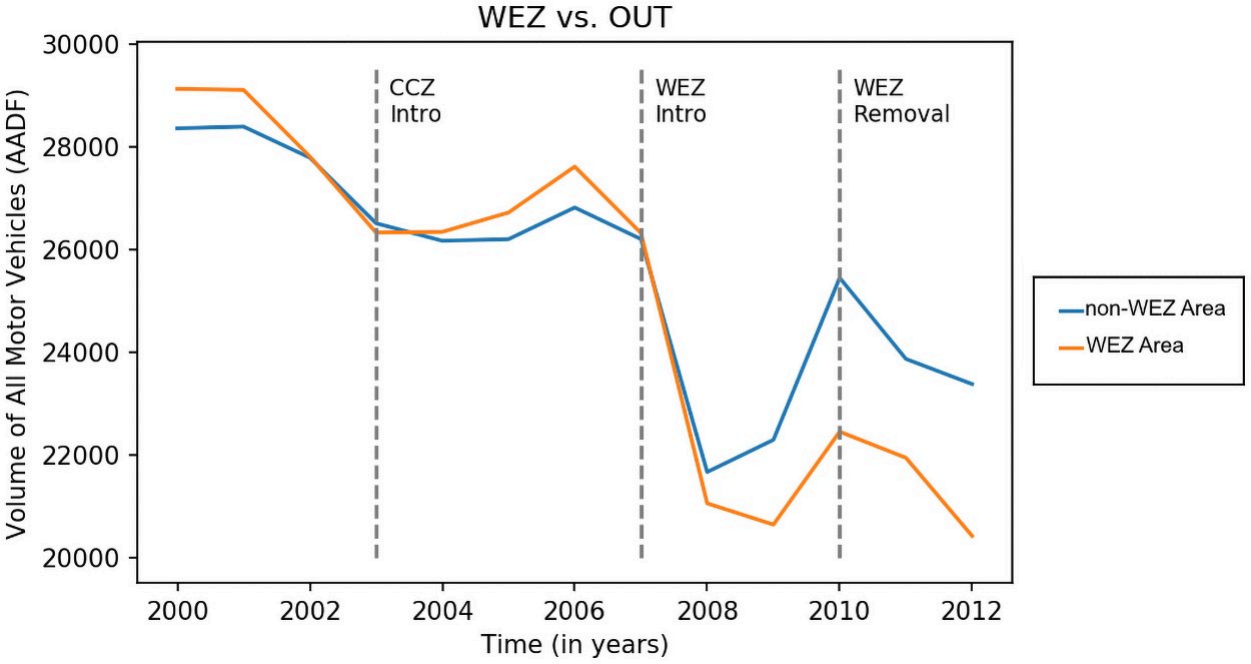
Annual average traffic flows in Greater London between 2000 and 2012 (source: AADF, DfT).

We use a difference-in-differences methodology (DiD) to identify causal responses to the introduction and the removal of the WEZ. We rely on the assumption that the WEZ reforms were not anticipated by individuals. As car ownership entail sunk costs including an important fixed upfront cost, we assume that car purchases and habits would not have changed until the financial incentives are implemented. Fig 2 indicates no clear anticipation behaviour of our groups, but this assumption is further tested in the Robustness Checks section. We also posit that the trend in the treatment and the control group would have been the same in the absence of the treatment [42]. We test the conditional parallel trend via a placebo test in section 6.4.

We specify two separate regressions for the introduction and the withdrawal of the CC in London. In this study, we prefer estimating separate difference-in-differences models for successive treatments that are the (i) implementation and (ii) removal of the CC rather than consider them jointly in a single model, because there could be effect heterogeneity for at least one of the treatments which could impair the identification strategy. The two main econometric specifications used in this study to assess traffic responses respectively for the introduction and removal of the WEZ are the following:

$$\begin{array}{c} Y_{it}=\mu +\alpha WEZ_{i}+\delta POST_{t}^{Intro}+\tau WEZ_{i}\cdot POST_{t}^{Intro}+\beta X_{it}+\phi_{i}+\gamma_{t}+\varepsilon_{it} \end{array}$$ (1)

$$\begin{array}{c} Y_{it}=\mu +\alpha WEZ_{i}+\delta POST_{t}^{Removal}+\tau WEZ_{i}\cdot POST_{t}^{Removal}+\beta X_{it}+\phi_{i}+\gamma_{t}+\varepsilon_{it} \end{array}$$ (2)

Where *Y*_*it*_ is the average annual traffic flow at count point *i* in borough *b* at time *t*, *WEZ*_*i*_ is a dummy variable equal to 1 if the observation is located in the WEZ and zero otherwise; *X*_*it*_ is a vector of location characteristics, namely the annual fuel retail price, borough population and employment rates at borough level; *μ* is the constant term; *ϕ*_*i*_ controls for count point fixed effects, *γ*_*t*_ controls for time fixed effects, and *ε*_*it*_ is the error term.

In this study, $POST_{t}^{Intro}$ is a dummy equal to 1 if the date *t* ≥ 2007, which is the year at which the WEZ was introduced, and zero otherwise. Similarly, $POST_{t}^{Removal}$ is a dummy equal to 1 if the date *t* ≥ 2010, which is the year at which the WEZ was removed, and zero otherwise.

We do not use transform outcomes into logs in the DiD specifications in order to keep the observations that are equal to 0: this may arise for some streets that can see no traffic at certain times, such as no cars, or no motorbikes.

Count point fixed effects control for the volume of traffic observed as it can be impacted by both road characteristics (*e.g.*, number of lanes) and borough characteristics (*e.g.*, population, rate of employment). Controlling for population density and employment rate controls whether the area is residential or more of an office district. We control for time trends to account for temporal traffic trends such as the general increase in bus patronage [36]. Fuel price controls are also added in the specification in order to account another determinant of the intensity of car usage.

Our coefficient of interest in the DiD estimation is *τ* which quantifies the effect of the treatment—that is the introduction or the removal of the WEZ—on the the treated roads that are located in the WEZ area. Following the findings from the literature, we can expect *τ* < 0 after the introduction of the WEZ since the implementation of the WEZ provides a financial incentive to drivers to shift to more sustainable transport modes that are exempt from the charge. Conversely, we expect a negative or potentially insignificant *τ* after the WEZ was removed since drivers might revert back to their pre-WEZ behaviour. However, financial incentives embedded in the removal remain small, which implies that the increase in car flows might be insignificant.

## 5 Data

To perform this analysis, we use Annual Average Daily Flow (AADF) data produced by the UK Department for Transport (DfT). This dataset records information on traffic levels and road characteristics for 2,991 count points in London over the years 2006–2012—including 991 points in the treated area—and totalling 12,827 observations. Each observation contains information for a given street for an average day of a given year, namely the number of vehicles travelling past a specific count point in both directions. Traffic flows are counted for each of the following transport modes: “cars and taxis”, “buses and coaches”, heavy goods vehicles (HGVs), light goods vehicles (LGVs), and the total four-wheeled vehicles (categorised as “all motor vehicles”).

Cars and taxis are not split in the dataset, which implies that we cannot separate the responses of each vehicle type to the introduction and removal of the WEZ in our analysis. We acknowledge that this category contains both treated (cars) and untreated (taxis) vehicles. S1 Table presents mode shares for London and shows that including the cars and taxis section is crucial as they represent the majority of vehicles on roads. Yet, the analysis of the incentives contained in WEZ policies for respectively cars and taxis imply that the presence of a joined group does not impair the quality of the insights provided in our analysis. The implementation of the WEZ generates negative financial incentives for cars; as such, we expect cars’ traffic flows to decrease for cars post-WEZ. However, taxis are not subject to these negative financial incentives: therefore, we expect the flow of taxis in streets to remain constant or to increase in case individuals substantially shift their modes of transport from cars to taxis post-WEZ. At first, expected effects could be ambiguous—however, we still expect a negative coefficient for the group “cars and taxis”. This implies then that we capture a lower bound effect, and this does not undermine our assessment of significant responses.

Fig 2 presents annual average traffic flows for all motor vehicles in both treated (WEZ) and control (non-WEZ) groups. The introduction of the WEZ appears to lead to a greater traffic decline in the treated group as traffic levels decreased more in the WEZ area. The WEZ removal appears to have caused a smaller traffic reduction in traffic in the treated group compared to the control group. These descriptive statistics indicate also that there is initial support in favour of the common trend hypothesis.

Finally, we complement the aggregate nature of our data by using Consumer Data Research Centre (CDRC) data. The data consists of raw traffic counts collected annually from different count points in London between 8 and 9 am. The raw traffic counts were carried out on roughly the same day every year which controls for seasonal trends and each observation has a specific date. We apply the model to this data and the results are presented in S5 and S6 Tables. We find that neither the introduction nor the removal had a significant effect on the volume of traffic. We attribute this to the inelastic nature of trips made during rush hour which are usually work related.

## 6 Results and discussion

### 6.1 Difference-in-means analysis

S2 and S3 Tables present difference-in-means results for the introduction and removal of the WEZ respectively. To do so, we calculate the average change between the pre and post treatment period in the control and treatment groups and differentiate the two to compute the difference-in-means. Estimates indicate that the WEZ led to a decline in traffic for all modes affected by the WEZ, which provides preliminary evidence in favour of the efficacy of the WEZ. The insignificant change in HGVs might exhibit inelastic demand.

### 6.2 Responses to the implementation of the WEZ

Estimates in S4 Table represent traffic responses after the WEZ was introduced. Results indicate a significant decrease in the number of vehicles for all modes subject to congestion charging, with the exception of HGVs. This confirms the initial difference-in-means results. The coefficient attached to cars and taxis is negative and significant, which indicates that the negative financial incentives embedded in the WEZ policy encouraged a considerable decrease in car use that the potential increase in taxis did not mitigate. In order to better understand the magnitude of responses, we calculate demand elasticities. To do so, we re-estimate the model in Eq (1) using natural logarithms of traffic flows. This allows the computation of percentage changes as follows: *exp*(*coefficient*) − 1 = *Changein*% (see Table 1).

**Table 1:** DiD estimates associated with the analysis of the introduction of the WEZ using logs.

	Total	Cars and Taxis	Buses and Coaches	LGVs	HGVs
	(1)	(2)	(3)	(4)	(5)
Post 2007 (Yes = 1)	-1.205***	-1.174***	-0.765***	-1.062***	-0.838***
	(0.013)	(0.014)	(0.033)	(0.019)	(0.028)
WEZ (Yes = 1)	-1.488***	-1.519***	-0.662***	-1.877***	-2.418***
	(0.077)	(0.082)	(0.200)	(0.114)	(0.166)
**WEZ & Post 2007 (Yes = 1)**	-0.048**	-0.057***	**-0.003**	-0.074**	0.090**
	**(0.020)**	**(0.021)**	**(0.052)**	**(0.030)**	**(0.043)**
**% Change**	**4.962%**	**5.817%**	**0.308%**	**7.696%**	**9.415**
Observations	10,378	10,378	10,106	10,376	10,321
R-squared	0.993	0.991	0.97	0.986	0.98

*Note*: Natural logs of AADF are used as the explanatory variable in Eq (1).

Standard controls were used in this analysis.

***, ** and * respectively indicate significance at the 1%, 5% and 10% levels.

Standard errors are in parenthesis. Source: DfT AADF data. Years: 2006–2010.

Estimates show that overall, motor vehicles flows decline by 4.9% in the Western Zone extension area after the extension of the congestion charge area. This is smaller than the value of 14% reported in previous studies [30] but remains comparable to the 5.6% decline in more recent reports [31]. Cars and taxis decreased by 5.1%. This is much lower than TfL’s (2008) estimate of -22% for cars and might be caused by the fact that taxis, that are exempted from constraint, saw their flow increase post-WEZ. Yet, our estimates tend to validate more previous studies’ conclusions [28, 30], namely that the Western expansion did not meet its target of a 13% reduction in total vehicle traffic.

We also find a 6.7% decrease in LGVs, which is similar to 7% estimate previously found [39]. Unlike LGVs, HGVs flows did not significantly vary after the introduction of the WEZ. This can be explained by the fact that HGVs are mostly used for commercial purposes and constitute a category of vehicles which tend to exhibit an inelastic demand [43].

We do not record any change in traffic volumes for buses and coaches post-WEZ implementation, which is unsurprising since they were exempt from the charge, and the increase in bus patronage have been accounted for in the trend and year fixed effects. In addition, the number of buses is determined by the timetables, not directly by passenger demand. More broadly, results suggest that the decline in traffic flows is smaller than the 30% decrease recorded after the London CC scheme was implemented in 2003. The reduced sensitivity might be explained by the fact that the WEZ covers a smaller zone compared to the 2003 CC zone. Thus, the smaller decline might have occurred since only a third of the traffic entering the zone was expected to pay the CC [44].

### 6.3 Responses to the removal of the WEZ

Estimates in Table 2 below show that the removal of the WEZ had no significant effect on overall traffic, which is in line with previous studies [31]. This validates even further the results in the literature as our results have more power and all our specifications account for fixed effects. In contrast, HGV traffic flows increase by a significant 20% post removal of the WEZ, which indicates that these vehicles are not sensitive to financial disincentives but significantly respond to financial incentives—in this case, a reduction in costs.

**Table 2:** DiD estimates from analysis for the removal of the WEZ.

	Total	Cars and Taxis	Buses and Coaches	LGVs	HGVs
	(1)	(2)	(3)	(4)	(5)
Post 2010 (Yes = 1)	-9,327.26***	-6,969.75***	-124.23***	-1,803.13***	-333.68***
	(546.35)	(472.37)	(23.33)	(112.00)	(51.87)
WEZ (Yes = 1)	-25,469.44***	-20,924.59***	364.51***	-4,330.80***	-1,790.11***
	(2,152.76)	(1,861.25)	(91.93)	(441.33)	(204.39)
**WEZ & Post 2010 (Yes = 1)**	**198.71**	**226.65**	**-15.03**	**-72.32**	117.84**
	**(490.23)**	**(423.85)**	**(20.93)**	**(100.50)**	**(46.54)**
Observations	12,377	12,377	12,377	12,377	12,377
R-squared	0.979	0.974	0.949	0.954	0.963

*Note*: The specification follows Eq (2) and includes annual employment rate and population controls, time trends, and time and borough fixed effects.

***, ** and * indicate significance at the 1%, 5% and 10% levels. Standard errors are in parenthesis.

Source: DfT AADF data. Years: 2007–2012.

Our results differ from the previous significant 14.5% increase in traffic volume found after the road pricing scheme in Milan got temporarily suspended [45]. As such, our results might be explained by the fact that the WEZ lasted longer than the CC schemes in Stockholm and Milan. For instance, the CC withdrawal occurred in Stockholm 6 months after its implementation, whereas the WEZ lasted substantially longer (4 years), leaving individuals a time window large enough to significantly change their travel attitudes in a way that would affect the determinants of car usage (*e.g.*, car ownership). However, after the WEZ was removed, individuals who wanted to shift back to their pre-WEZ travel habits had to face new costs generated by the car ownership and maintenance (*e.g.*, insurance costs).

### 6.4 Robustness checks

#### 6.4.1 Placebo test

We compute placebo tests to verify the validity of the common trend assumption [46]. We chose to not use a lags and leads specification as a robustness check because of the existence of other little events for some years (see Fig 1) that would have potentially biased the results. The aim of this test is to show that pre-treatment data presents a clear trend, which can then be applied to the post-reform period. Placebo tests involve checking the existence of a treatment effect on traffic by changing the date of the reform. For the WEZ implementation (respectively removal), the analysis will be repeated using only pre-treatment (respectively post-treatment) years and an arbitrary treatment year will be selected from these. The specification follows Eq (1) with the implementation date being now *T*_*i*_ = 2006 and *T*_*r*_ = 2011. In other words, since the treatment has not been dispensed, we do not expect to find an effect on the treated group post-treatment.

Table 3 and S7 Table present placebo tests results respectively for the introduction and removal of the WEZ. Estimates are insignificant for all vehicle types subject to changes in road pricing, which is in line with the fact that the parallel trend hypothesis is verified and it is the treatment that is at the source of the response and that effects are not resulting from general trend effects.

**Table 3:** Placebo test estimates—Removal of the WEZ.

	Total	Cars and Taxis	Buses and Coaches	LGVs	HGVs
	(1)	(2)	(3)	(4)	(5)
Post 2007 (Yes = 1)	-2,245.21***	-1,429.15***	-47.42***	-602.60***	-86.92***
	(290.64)	(262.33)	(11.80)	(56.74)	(26.90)
WEZ (Yes = 1)	-26,193.16***	-18,959.19***	167.04	-6,531.59***	-1,571.23***
	(4,355.76)	(3,931.53)	(176.80)	(850.36)	(403.16)
**WEZ & Post 2009 (Yes = 1)**	**-180.04**	**-140.49**	47.18*	**-34.06**	**11.76**
	**(664.29)**	**(599.60)**	**(26.96)**	**(129.69)**	**(61.49)**
Observations	6,529	6,529	6,529	6,529	6,529
R-squared	0.985	0.981	0.968	0.969	0.975

*Note*: The period of analysis was from 2008 to 2010 with the pseudo treatment in 2009.

The controls used were the same as those discussed in Eq (1).

***, ** and * respectively indicate significance at the 1%, 5% and 10% levels. Standard errors are in parenthesis.

Source: DfT AADF data. Years: 2017–2012.

In addition to this placebo test using different cutoff dates, we run additional regressions and change the count points that are to be included in the treatment area. We set the treatment a bit westward in a way that includes the borough of Hammersmith and Fulham and also find null results for both the implementation and removal of the WEZ. This further confirms the robustness of the results. Finally, additional regressions have been performed using a two period DiD for respectively the introduction and removal, which yield similar results compared to what is obtained in Tables 1 and 2. This implies that the results are correctly identified by the multiple periods difference-in-differences methodology used in the present study.

#### 6.4.2 Buffer test

One of the underlying DiD assumptions (see Section 4) is that treated units do not interact with untreated units. To verify this hypothesis, we perform a buffer test following [31]. To implement the buffer test, we perform the previous analysis following Eqs 1 and 2 while removing count points within 500m of the WEZ boundary. Table 4 presents the buffer test results after the WEZ was introduced. This test indicates that our results are robust since the DiD estimates are of a similar magnitude and sign.

**Table 4:** Buffer test DiD estimates—Introduction of the WEZ.

	Total	Cars and Taxis	Buses and Coaches	LGVs	HGVs
	(1)	(2)	(3)	(4)	(5)
Post 2007 (Yes = 1)	-5,530.04***	-3,630.00***	-78.75***	-1,421.96***	-242.77***
	(398.79)	(355.03)	(16.39)	(88.97)	(38.37)
WEZ (Yes = 1)	-20,817.18***	-13,497.84***	422.71***	-6,717.08***	-1,794.27***
	(2,499.95)	(2,225.62)	(102.75)	(557.71)	(240.51)
**WEZ & Post 2007 (Yes = 1)**	-1,742.06**	-1,380.51*	**24.79**	-371.93*	**78.97**
	**(851.26)**	**(757.85)**	**(34.99)**	**(189.90)**	**(81.90)**
Observations	9,897	9,897	9,897	9,897	9,897
R-squared	0.983	0.978	0.957	0.958	0.97

*Note*: Here, a 500m buffer on either side of the WEZ boundary is excluded from the analysis to eliminate interaction between the treated and control units. The standard controls are included.

***, ** and * respectively indicate significance at the 1%, 5% and 10% levels. Standard errors are in parenthesis.

Source: DfT AADF data. Years: 2006–2010.

### 6.5 Discussion

Results from this study have thus far suggested that the introduction of a CC scheme appears to lead to a significant and substantial reduction in the volume of traffic for most modes of travel, with the largest response being recorded for cars. An important finding is that the introduction and the removal of a congestion charge in a given area do not have symmetrical effects. More precisely, removing a CC appears to have no significant effects on traffic. This suggests that individuals, to some extent, maintain the travel behaviours they adopted under the presence of a congestion charge. These results show that a policy which temporarily implements a CC can cause a lasting shift in travel behaviour decisions under the condition that the withdrawal is unanticipated. This confirms previous evidence from Stockholm [40] that the “temporary” implementation of the CC led to a reduction in traffic that persisted even when it was removed. These results generate policy implications since despite their subsequent removal, CC zones are expected to continuously reduce traffic through making driving less attractive.

## 7 Conclusion

This paper quantifies traffic responses after the introduction and the removal of a congestion charge on traffic volume using a difference-in-difference (DiD) methodology. Results indicate that the introduction of the WEZ led to significant responses, with a 4.9% decline in the total volume of traffic and a 5.1% decline in car traffic in the WEZ area. Heavy Goods Vehicles (HGVs) did not significantly change their travel patterns, which might be explained by their price inelastic demand for road use. Further analysing responses to the removal of WEZ show that responses to the implementation and withdrawal of the WEZ are not symmetrical. Estimates recording changes in traffic volumes are insignificant for all vehicles subject to the change in policy. The withdrawal of a congestion charge increases financial incentives to use one’s car, yet this incentive might be marginal and not sufficient for individuals to increase their propensity to change their car use to an extent that makes a substantial difference in terms of traffic flows. Therefore, the change in behaviour after a congestion charge is implemented is likely to be sustained even after this policy is withdrawn. Results are robust to different specifications, placebo tests and buffer tests.

More broadly, our results indicate that congestion charging lead to a persistent change in aggregate travel demand levels even after the removal of the CC. Our results provide grounds for policy intervention in favour of the implementation of a congestion charge since they tend to have lasting effects that persist even after its withdrawal.

## Supporting information

S1 Table Road share in London—Source: DfT AADF data. (TIF)

S2 Table Difference-in-mean estimates for the introduction of the WEZ. (TIF)

S3 Table Difference-in-mean estimates for the removal of the WEZ. (TIF)

S4 Table DiD estimates from analysis of the introduction of the WEZ. (TIF)

S5 Table DiD estimates from analysis of the introduction of the WEZ using CDRC data. (TIF)

S6 Table DiD estimates from analysis of the removal of the WEZ using CDRC data. (TIF)

S7 Table Results from the placebo test for introduction of the WEZ. (TIF)
