# Supplementary figures and images for: Quantifying responses to changes in the jurisdiction of a congestion charge: A study of the London western extension
Source: PLoS One. 2021 Jul 1;16(7):e0253881. doi: 10.1371/journal.pone.0253881 (PMC8248659; doi:10.1371/journal.pone.0253881)

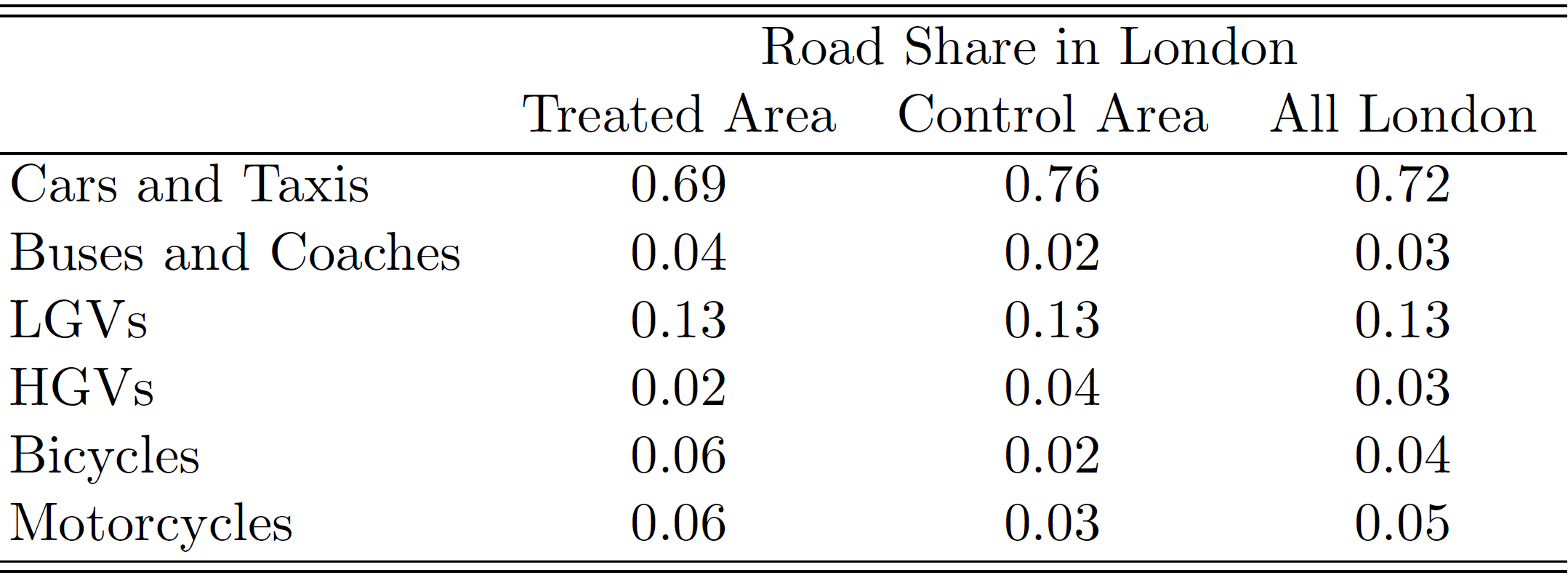

Supplement: S1 Table — (TIF) [file pone.0253881.s001.tif]

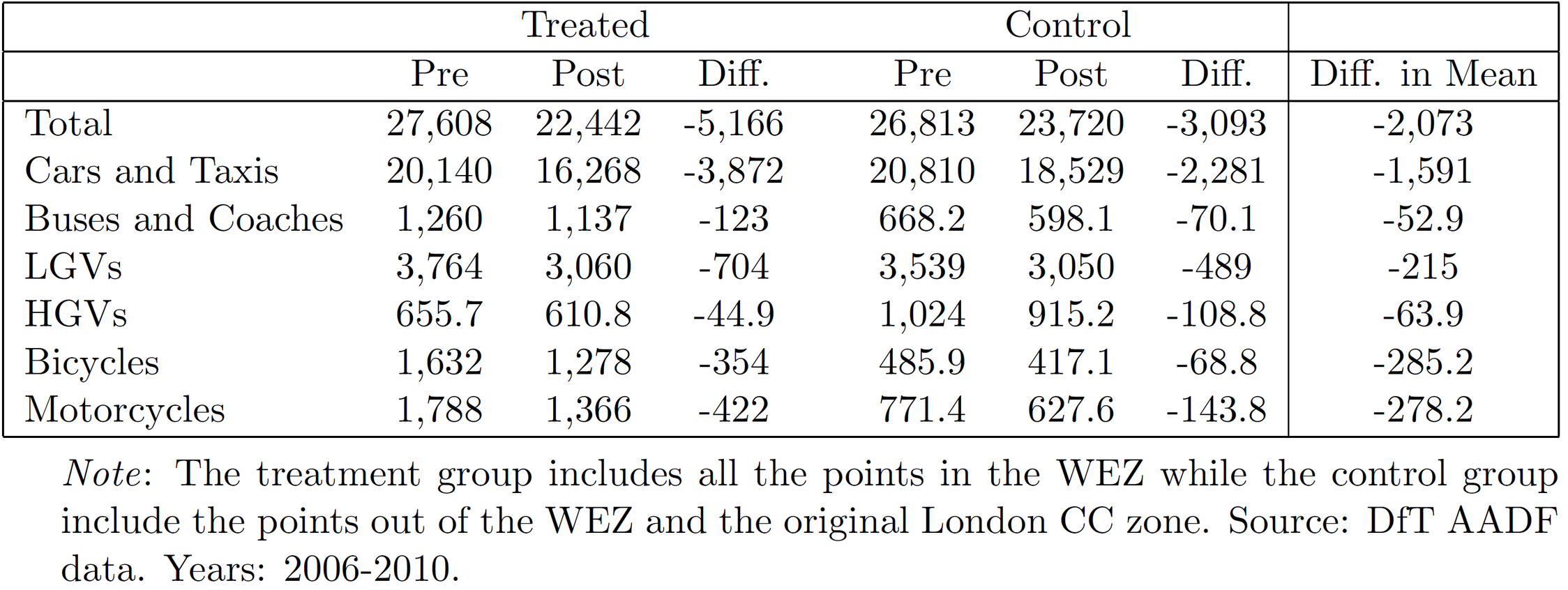

Supplement: S2 Table — (TIF) [file pone.0253881.s002.tif]

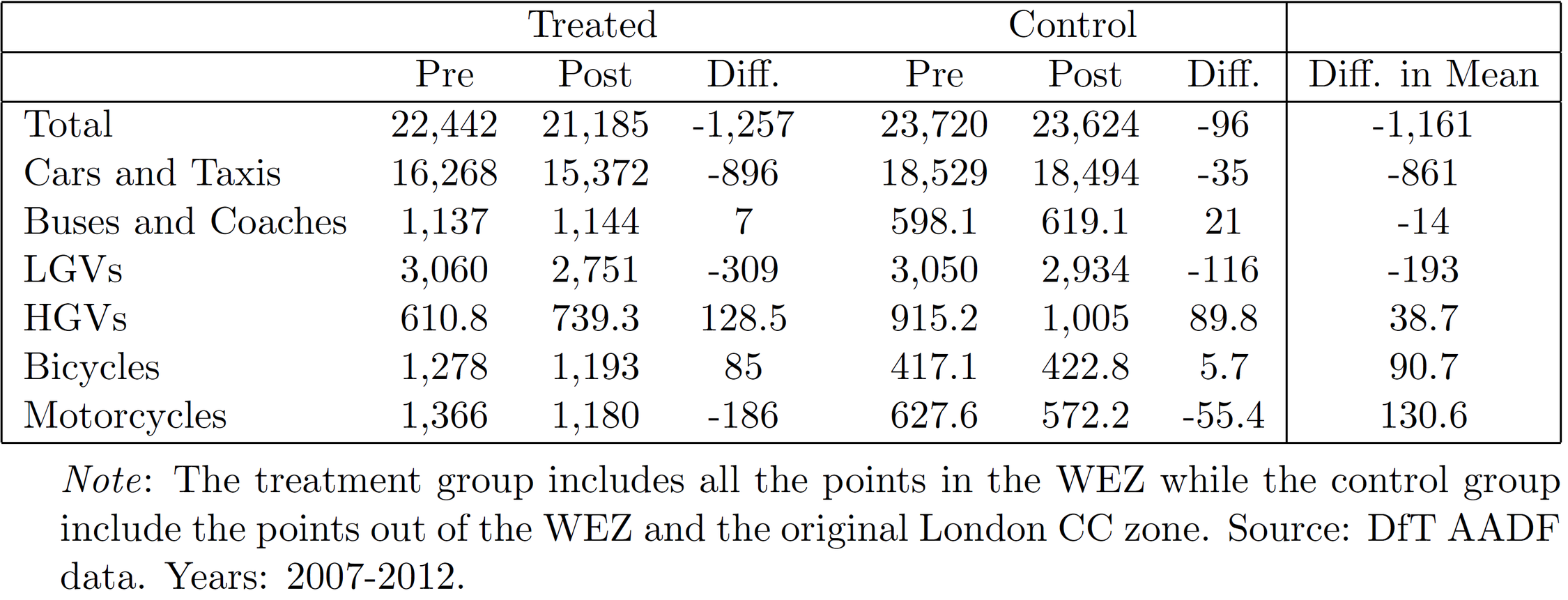

Supplement: S3 Table — (TIF) [file pone.0253881.s003.tif]

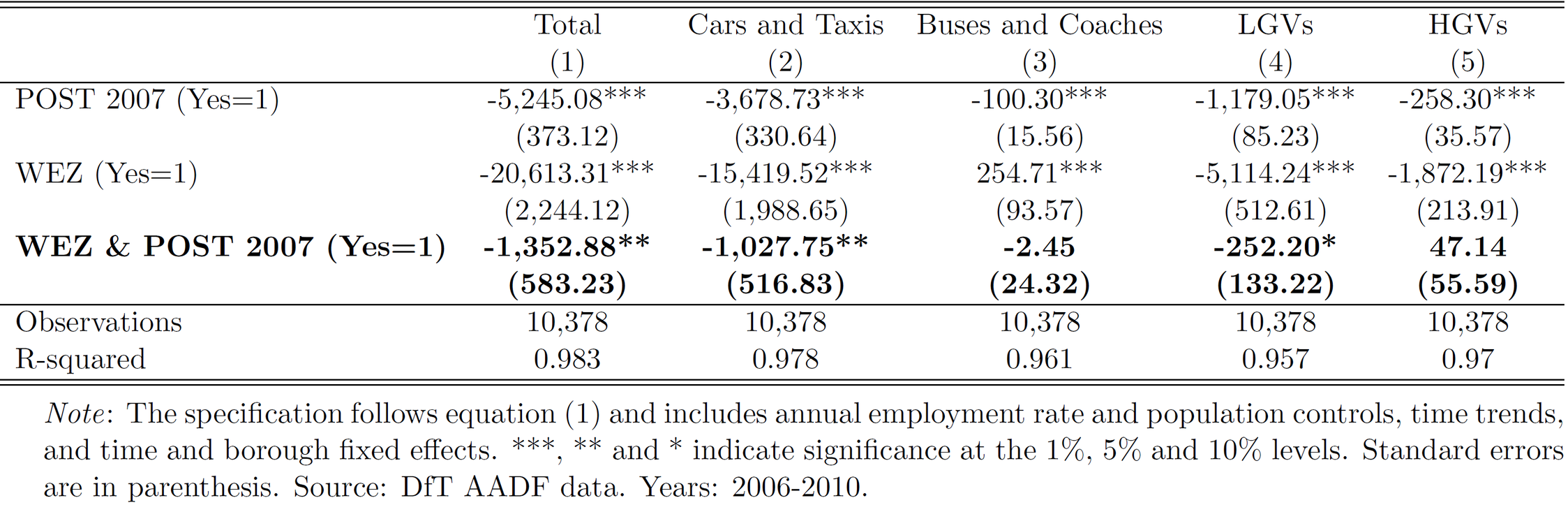

Supplement: S4 Table — (TIF) [file pone.0253881.s004.tif]

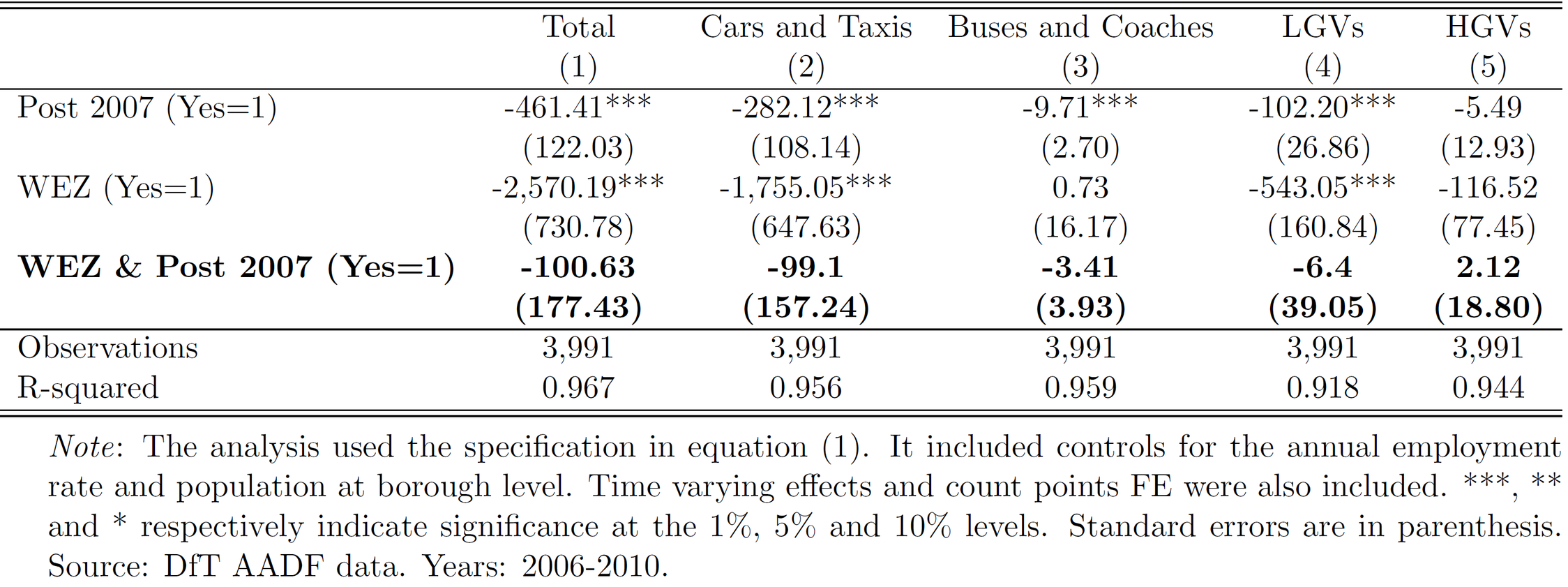

Supplement: S5 Table — (TIF) [file pone.0253881.s005.tif]

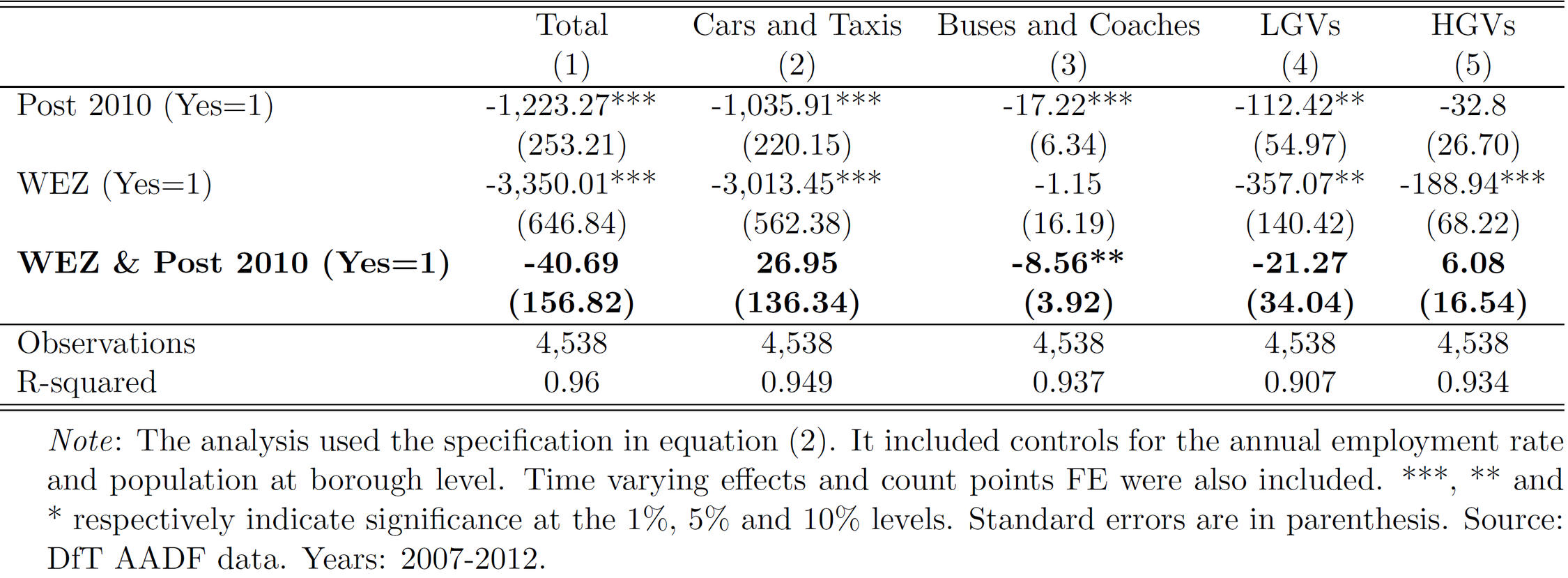

Supplement: S6 Table — (TIF) [file pone.0253881.s006.tif]

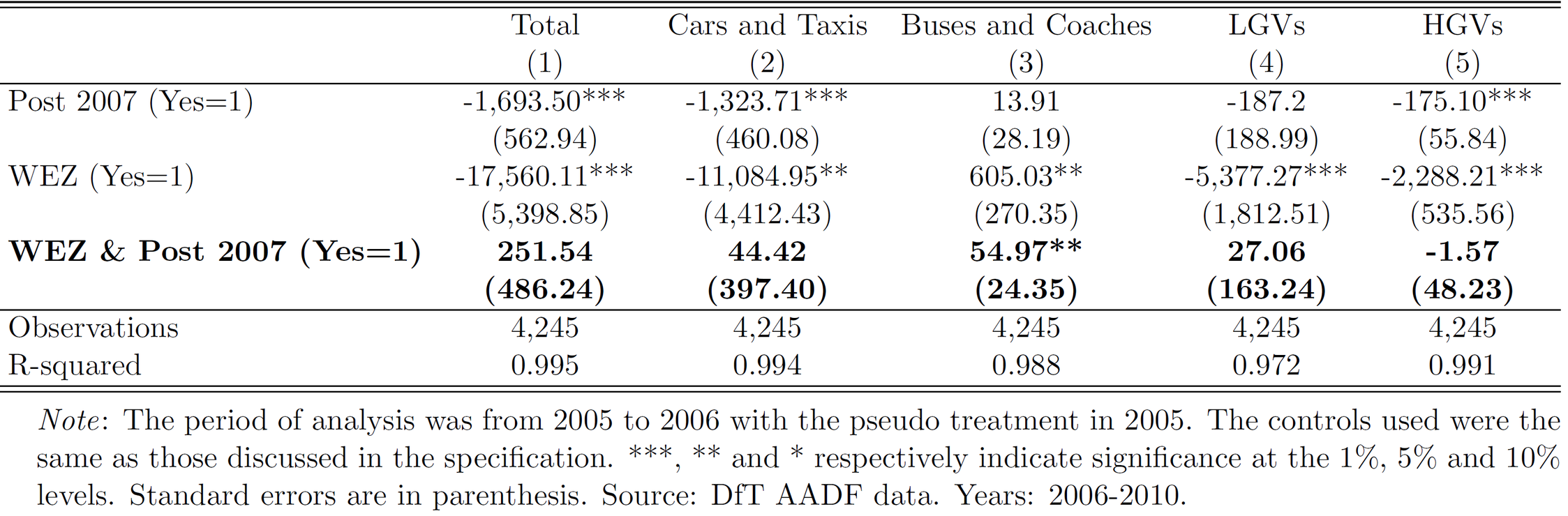

Supplement: S7 Table — (TIF) [file pone.0253881.s007.tif]
